# The European Masters Degree in Disaster Medicine (EMDM): A Decade of Exposure

**DOI:** 10.3389/fpubh.2014.00049

**Published:** 2014-05-21

**Authors:** Francesco Della Corte, Ives Hubloue, Alba Ripoll Gallardo, Luca Ragazzoni, Pier Luigi Ingrassia, Michel Debacker

**Affiliations:** ^1^Research Center in Emergency and Disaster Medicine and Computer Science Applied to Medical Practice (CRIMEDIM), Novara, Italy; ^2^Research Group in Emergency and Disaster Medicine (ReGEDiM), Vrije Universiteit Brussel, Brussels, Belgium

**Keywords:** disaster medicine, disaster medicine education, masters degree, outcome, certification

Disasters often overwhelm the capacity of communities and health systems to deal with the massive number of victims and the scarcity of available resources (1, 2). In crisis situations, health professionals are required to perform tasks that fall outside their routine work (3), such as mass casualty triage and management of specific populations such as refugees and internally displaced people. Education in disaster medicine is therefore crucial to provide professionals with the necessary skills, knowledge, and attitudes to adequately respond to disaster situations.

Even though courses in disaster medicine have been shown to be beneficial at the undergraduate level (3), master degrees offer the advantage of tailoring training programs to the needs of advanced professionals. As a result, graduates have a higher level of professional competence and better employment opportunities (4). Since 2000, the European Masters Degree in Disaster Medicine (EMDM) has been providing health workers with the specific competencies necessary to efficiently address and coordinate the medical response during disasters locally, nationally, and internationally.

## The Course

The EMDM is an advanced Masters of Science aimed at all health professionals involved in medical disaster management. It is jointly organized by the Research Center in Emergency and Disaster Medicine of the Università del Piemonte Orientale (Italy) and the Vrije Universiteit Brussel (Belgium), together with other institutions such as the Centre for Teaching and Research in Disaster Medicine and Traumatology (University of Linköping, Sweden), the Center for Disaster Medical Sciences (University of California at Irvine, USA), and the University Hospitals of Geneva (Switzerland). It is also supported by other relevant international organizations such as the World Health Organization (WHO), the International Committee of the Red Cross (ICRC), and the European Society for Emergency Medicine (EuSEM).

The EMDM Masters program supports the development of disaster medicine at the academic level with the intent of creating health workers with a high-level of professionalism, ready to be deployed with governmental and non-governmental organizations. Specifically, upon completion of the Masters, graduates are expected to be able to:

- Define the main academic, legal, and ethical principles associated with disaster medicine.
- Assess the impact of disasters on the health system, analyze risks, and develop primary prevention plans.
- Manage medical responses in diverse disaster situations.
- Organize education and training in disaster medicine.
- Develop research projects in disaster medicine.

The educational structure is based on a multidisciplinary and competency-based approach. Learning takes place through a combination of traditional and innovative educational methods. The didactic approach utilizes a self-directed e-learning curriculum in addition to 2 weeks of classroom education. In the e-learning phase, students are involved in virtual team-working exercises and learn at their own pace, under the guidance of tutors who are EMDM faculty members. During the classroom work, students meet peers and faculty, attend lectures, participate in debates, and engage in complex simulations. One of the main strengths of our Masters program is the presence of international faculty members with field experience in natural, technological, terrorist, and humanitarian large-scale emergencies. The Masters degree is awarded upon the successful completion of an online proctored examination and upon the defense of an original thesis dissertation, with the expectation of publication in a peer-reviewed journal.

## Outcomes and Critical Reflections

At this time, the EMDM Masters program has provided fundamentals of research in disaster medicine to almost 400 professionals. The geographical origins of students are summarized in Table 1.

**Table 1:** Nationality of EMDM students*.

			*n* = 397
Albania	2	Lebanon	4
Algeria	3	Libia	2
Argentina	1	Malaysia	4
Australia	7	Malta	3
Austria	1	Mexico	4
Bahrain	6	Namibia	1
Belgium	13	Netherlands	3
Brasil	1	New Zealand	2
Cameroun	2	Nicaragua	1
Canada	19	Nigeria	5
China	4	Oman	4
Colombia	3	Pakistan	8
Congo Rep.	1	Peru	1
Croatia	1	Philippines	3
Cyprus	1	Portugal	9
Dominican Rep.	1	Qatar	8
Ecuador	1	Romania	3
Egypt	10	Saudi Arabia	21
Finland	3	Senegal	1
France	2	Serbia	1
Georgia	1	Singapore	5
Germany	16	Solomon Islands	1
Greece	17	South Africa	6
Hong Kong	1	Spain	4
Hungary	2	Sri Lanka	1
India	7	Sudan	16
Indonesia	1	Sweden	3
Iran	3	Switzerland	2
Iraq	3	Syria	1
Ireland	9	Trinidad and Tobago	1
Israel	2	Tunisia	1
Italy	62	Turkey	5
Japan	3	UAE**	7
Jordan	3	Uganda	1
Kenya	2	UK***	14
Korea	1	USA****	23
Kosovo	1	Yemen	2

^*^Referred to the editions from 2000 to 2013.

^**^UAE United Arab Emirates.

^***^UK United Kingdom.

^****^USA United States of America.

In an unpublished survey conducted in 2012, a total of 207 out of the 288 students who attended the first 10 EMDM courses (72% response rate) reported the outcomes of their participation. Most students stated that the EMDM program had helped them not only to achieve their short (48%) and long term goals (55%) but also to gain new perspectives (42%). Fifty-six percent had acquired new professional prospects and 60% affirmed that the course had offered them opportunities to meet leading experts in their professional fields. Interestingly, 30% had been offered a higher rank/responsibility in their institutions and 15% had progressed to a better job. Finally, 51% ranked the EMDM experience as excellent, 34% very good, and 12% interesting.

In the EMDM educational program, the e-learning platform plays a central role as a unique means of communication and information exchange. Electronic learning has been widely demonstrated to represent a valid and innovative educational method for postgraduate students (5). By using e-learning, scholars worldwide can simultaneously benefit from courses tailored around their own commitments, interact with peers and faculty members either in synchronous or asynchronous ways, and receive support in near real time (6). In addition, hyperlinking allows users to rapidly access various information sources, resulting in the acquisition of more knowledge in a shorter time (5). Some of our students reported feeling overwhelmed by having simultaneously to use the virtual learning platform and study the didactic material. To address these concerns, the organizing universities improved the technical assistance for possible problems related to the e-learning platform, introduced individual faculty tutors (2006) to assist students as needed, and implemented videoconferences to reinforce learning (2010).

The 2-week on campus course is designed to enhance the learning process and offers unique opportunities to strengthen social relationships. Discussion sessions, in an international and multicultural community of students and faculty, promote expansive and creative thinking. In fact, one of the most important tenets of the EMDM program is the recognition of each student’s prior experience. Participants actively contribute to the learning process by sharing their experiences, opinions, and feelings. These interactions allow students to develop confidence in their own abilities while contributing to a collaborative and stimulating learning environment.

Because many disasters are sudden events, training in real disaster situations can rarely be implemented. Virtual simulations offer opportunities to train health professionals in the management of complex disasters through a variety of virtual scenarios without economic, temporal, or organizational limitations (6). Simulations also offer the opportunity to apply the concepts of the Masters course to situations, which may be encountered by students in the future.

Some studies have highlighted the need to make disaster medicine more evidence-based (2, 7–9). In line with this statement, the EMDM community is particularly concerned with the promotion and development of disaster medicine as an academic discipline. The first EMDM programs demonstrated that students needed advanced education in research methodology, data collection/analysis, and scientific writing. Unfortunately, only 23% of respondents to our survey succeeded in publishing their Masters dissertation. To overcome this deficit, a dedicated program addressing the topic of research applied to disaster medicine was incorporated into the current curriculum. A specific component of the curriculum included the presentation and evaluation of the rigorous approach for dissertation proposals. An experienced faculty mentor was assigned to each student for dissertation development. It is encouraging that requests for doctoral opportunities in disaster medicine continue to increase.

Several graduates maintain contact with the disaster community through the EMDM Alumni association. This organization was created in 2005 with the dual purpose of promoting the study and practice of disaster medicine while assisting members of the EMDM network in the development of their professional careers. Rigor can be achieved by offering members continuous educational and dynamic opportunities for shared learning. The Alumni association also serves to promote collaborative initiatives and recruit individuals for international multidisciplinary projects.

The EMDM program is now in its fourteenth year. From the initial educational efforts, the mission of the organizations has been to promote a global collaboration through a flexible learning environment combining traditional and innovative approaches to disaster medicine education. The course has allowed students to achieve their goals, widen their professional perspectives and advance their careers. Building on the encouraging feedback received from former students, the EMDM community will continue to train health professionals, offering the highest standards of education in disaster management as well as new opportunities for employment and research.

## Conflict of Interest Statement

The authors declare that the research was conducted in the absence of any commercial or financial relationships that could be construed as a potential conflict of interest.
